# Taxonomic revision of the Mesoamerican genus *Spathacanthus* (Justicieae, Acanthoideae, Acanthaceae)

**DOI:** 10.3897/phytokeys.144.46929

**Published:** 2020-03-17

**Authors:** Mireya Burgos-Hernández, Gonzalo Castillo-Campos

**Affiliations:** 1 Programa de Botánica. Colegio de Postgraduados. Carretera México-Texcoco km 36.5, Montecillo, Texcoco 53230, Estado de México, México Colegio de Postgraduados Estado de México Mexico; 2 Red de Biodiversidad y Sistemática. Instituto de Ecología, A.C. Carretera antigua a Coatepec 351, Xalapa 91073, Veracruz, México Instituto de Ecología Xalapa Mexico

**Keywords:** conservation, distribution, endemism, phylogeny

## Abstract

*Spathacanthus* is a Mesoamerican genus that occurs in tropical and temperate regions from southern Mexico to Costa Rica; its taxonomy has not been updated for two decades. In view of the fact that a new species has been discovered and that the interspecific affinities in this genus have not been addressed to date, the present study aims to revise the genus *Spathacanthus*. Specimens of plants of this genus collected from across the distribution range and deposited in herbaria and digital databases were reviewed. In parallel, a cladistic analysis was carried out, based on morphological characters in order to examine relationships between species. Four species of *Spathacanthus* were recognised: one endemic to Costa Rica, another micro-endemic to Veracruz in Mexico, one more restricted to the forests of Mexico and Guatemala and the last one more widely distributed. Reflecting the previously limited knowledge of the group, many of the specimens that we studied had been misidentified. A key to differentiate these species is provided, supplemented with photographs, drawings and other illustrations, morphological descriptions, synonymy and ecological data. Results, presented here, extend the distribution range of some taxa and a distribution map is presented. The cladistic analysis recovered the genus as monophyletic, showing that *S. hoffmannii* and *S. hahnianus* are sister taxa and *S. magdalenae* was found to be more closely related to *S. parviflorus*. These plants are vulnerable to degradation and habitat loss.

## Introduction

The family Acanthaceae comprises more than 4,000 species and some 230 genera widely distributed throughout the world. They mostly thrive in tropical and subtropical areas, with the Indo-Malay, African (including Madagascar), South American and Mexican-Central American regions as primary diversity centres. The species of this family thrive in virtually all intertropical habitats, except for high-mountain areas (above 3,000 metres a.s.l.).

Mesoamerica is one of the seven American biodiversity hotpots where species face a high risk of extinction, associated with accelerated deforestation and global warming (Myers et al. 2000; Malcolm et al. 2006). Consequently, an enormous portion of the Mesoamerican flora may well become extinct before species can be described, much less characterised taxonomically and in terms of evolutionary relationships.

Mexico is a major centre of species richness, diversity and endemism for the family Acanthaceae (Daniel 2007), which ranks fifteenth in terms of floristic diversity in the country. Acanthaceae represent 1.6% of the total floristic richness of Mexico (Villaseñor 2016), with around 400 species (47 genera) of herbs, shrubs and trees; of these, 58.8% are endemic to Mexico (Villaseñor and Ortiz 2014), while seven of the 38 native genera documented in Mexico are also endemic to this country. Due to high species richness, no study has covered the entire family and new species are still being discovered (Daniel 2007).

*Spathacanthus* Baill. is a small genus of shrubs and trees; plants are distinctive amongst Mesoamerican Acanthaceae by having very large capsules and seeds, both of which are amongst the largest known in the family. The genus comprises four species distributed in moist to wet forests of Mexico and Central America. In Mexico, the genus is represented by three native species. Two of them were treated by Daniel (1995) – *Spathacanthus hahnianus* Baill. and *S. parviflorus* Leonard – and also inhabit Guatemala, with *S. hahnianus* reaching Honduras. *Spathacanthus magdalenae* Cast.-Campos was recently discovered in a riparian forest in Veracruz (Castillo-Campos et al. 2013). Meanwhile, *Spathacanthus hoffmannii* Lindau is restricted to Costa Rica (Lindau 1895a). Although currently it is accepted that the genus belongs to subfamily Acanthoideae, tribe Justicieae, *Pseuderanthemum* lineage (McDade et al. 2000), its generic relationships have not been resolved yet and the correct placement of the genus *Spathacanthus* has not been thoroughly assessed.

In addition to issues at the generic level, there is only one previous taxonomic treatment of the entire genus (Daniel 1999). The discovery of a fourth species (Castillo-Campos et al. 2013) clearly calls for re-assessment of the genus; also, interspecific affinities have yet to be addressed. Therefore, the present study aims to provide an updated taxonomic revision of the genus *Spathacanthus*, particularly in Mexico, where three of our species occur.

## Methods

This study was based primarily on herbarium specimens deposited in the following herbaria: BIGU, CHAPA, CR, ENCB, IEB, IBUG, MEXU, TEFH, USJ and XAL, as well as field observations of living plants. In total, 97 *Spathacanthus* specimens were examined. Digital specimens from the herbaria at CAS, DS, DUKE, F, K, MO, NY, TEX, US and UT were also consulted, all of them available via the TROPICOS (http://www.tropicos.org) and JSTOR Global Plants (https://plants.jstor.org/collection/TYPSPE) websites. Herbarium acronyms follow Thiers (2019). Protologues and type material of all species and synonyms involved were consulted. This allowed verifying or, where appropriate, re-identifying specimens from morphological features. We used a stereomicroscope (Stemi 2000-C, Carl Zeiss, Cd. Mx., Mexico) to study morphological variations amongst plants of all four species. New descriptions prepared for each species reflect the careful comparative work undertaken.

Specimens were reviewed using the dichotomous keys, descriptions and images provided by Daniel (1995, 1999) and Castillo-Campos et al. (2013). Additionally, all of the major floristic works for Mexico, Guatemala, Honduras and Costa Rica were reviewed (Baillon 1891; Lindau 1895a,b; Leonard 1937; Standley 1938; Standley and Steyermark 1974; Gibson 1974; Durkee 1986; Daniel 1993, 2010; Daniel et al. 2012).

The descriptions, newly presented here, include taxonomic and nomenclatural synonyms of each taxon, along with phenological information, habitat, distribution, elevation and vegetation types. The names of the species were verified on TROPICOS, The International Plant Name Index (https://www.ipni.org/) and Catalogue of Life 2020 Annual Checklist (http://www.catalogueoflife.org/col). Endemism was determined according to the descriptions in specialised studies, floristic listings, distribution data as reported in international databases (IPNI, TROPICOS, Global Biodiversity Information Facility (https://www.gbif.org/)) and information from herbarium specimens. A species was considered endemic if its distribution range is restricted to a given territory and as micro-endemic if exclusive to a limited site within a country. Distribution maps were prepared using geographic data via ARCGIS v.10.2.2 (ESRI 2014), based on the collection data compiled from herbarium specimens.

We also conducted a cladistic analysis, based on morphological characters as a first approach to explore relationships amongst species. We examined all four species of *Spathacanthus* as the in-group, plus *Odontonema callistachyum* Kuntze and *Odontonema cuspidatum* (Nees) Kuntze, as out-groups. Both genera – *Odontonema* and *Spathacanthus* – have been recognised as being the closest American relatives within the *Pseuderanthemum* lineage, in the tribe Justicieae (McDade et al. 2000). Based on the work of Daniel (1999) and after a detailed review of herbarium specimens, morphological characters derived from traditional external morphology were selected to build a matrix of morphological traits for cladistic analysis. A total of 15 binary and multistate characters were scored; the character by taxon matrix was built in MESQUITE v.3.2 (Maddison and Maddison 2017).

Character definitions and states are given in Appendices 1 and 2. A maximum-parsimony analysis (MP) was performed using PAUP* v.4.0a (build 165) (Swofford 2002); to this end, an exhaustive search was conducted involving 15 unordered characters given equal weight; multistate taxa were interpreted as polymorphisms. A total of 1000 replicates were carried out, auto-increased by 100, collapsing zero-length branches and with MulTrees in effect. The shortest trees were saved and a majority-rule consensus tree was produced. Statistical branch support was determined with 1000 non-parametric bootstrap (BS) iterations each with ten replicates, saving ﬁve trees per replicate. In addition, a Jackknife analysis (JK) was carried out with a 50% deletion and a full heuristic search; groups with a frequency > 50% were retained, with 1000 replicates and using the TBR branch-swapping algorithm. Finally, the tree was visualised with FIGTREE v.1.4.

## Results

### Taxonomic treatment

#### Spathacanthus

Taxon classificationPlantaeLamialesAcanthaceae

Baill. Hist. pl. 10: 444. 1891

FCC852AE-B21D-549B-80CE-BAC10FCB1811

##### Type.

*Spathacanthus hahnianus* Baill.

##### Description.

Small trees or shrubs, highly branched. Stems sometimes with conspicuous lenticels. Leaves opposite, sessile, subsessile to petiolate, entire, blade usually elongated, oblong or elliptical, membranous. Inflorescence terminal, cymose in the form of a thyrse, with opposite branches, consisting of dichasia or monochasia when one of the buds aborts, sometimes axillary, sessile to pedunculate, subtended by a bract; bracts opposite, small, green, entire. Flowers large, few, subsessile to pedicellate, pedicels usually enlarged in fruit; 2 homomorphic bracteoles; calyx green or yellowish, spathaceous, bilabiate equally or unequally divided into 2 segments; anterior segment with 2 fused lobes, apically entire to bifid; the posterior segment with 3 fused lobes, apically entire to trifid; corolla white or yellow, externally glabrous; tube expanded distally into a distinct throat; corolla with upper lip deeply bilobed, lower lip deeply trilobed; corolla lobes imbricate in bud. Stamens 4, didynamous, adnate at base of corolla throat; anthers monothecous or dithecous, if dithecous, then thecae equal in size, parallel, equally inserted, lacking basal appendages; anterior pair dehiscing towards upper lip, posterior pair dehiscing towards lower lip; staminodes lacking. Style glabrous, included in corolla tube; stigma bilobed, lobes equal. Capsule stipitate, large, glabrous, woody, green when immature, brown when dry. Seeds 4, homomorphic, flattened, glabrous.

**Figure 1.**
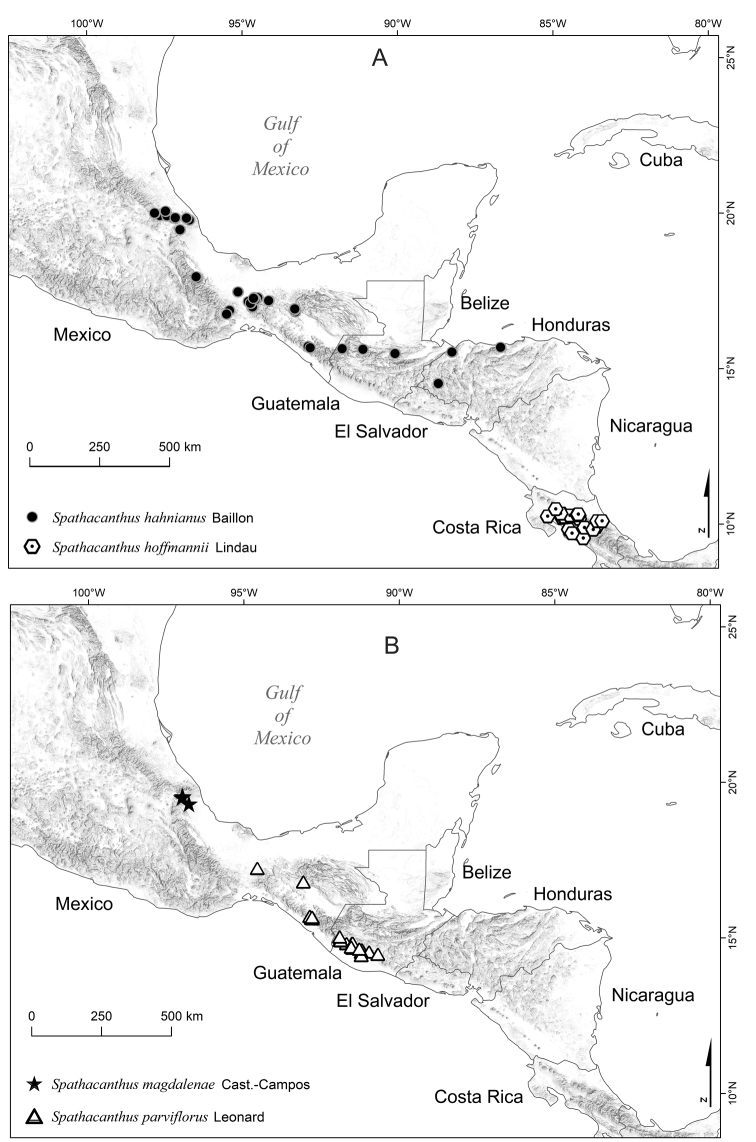
Distribution of the Mesoamerican genus *Spathacanthus* Baillon. **A** Distribution of *S. hahnianus* and *S. hoffmannii***B** distribution of *S. magdalenae* and *S. parviflorus*.

The genus *Spathacanthus* consists of four species occurring in humid tropical forests to temperate forests of south-eastern Mexico and Central America (Fig. 1A, B). The genus is easily differentiated from other Acanthaceae genera by the spathaceous calyx divided into 2 segments. The extremely large fruits are also distinctive amongst Neotropical Acanthaceae.

### Key to *Spathacanthus* species

**Table:** 

1	Calyx divided into 2 equal segments at anthesis	**2**
–	Calyx divided into 2 unequal segments at anthesis	**3**
2	Leaves subsessile, rarely shortly petiolate; calyx green before fruiting; restricted to Costa Rica	***Spathacanthus hoffmannii***
–	Leaves petiolate, petiole 10–50 mm long; calyx yellowish before fruiting; restricted to Mexico	***Spathacanthus magdalenae***
3	Corolla white, 25–30 mm long, throat 2–6 mm in diameter near midpoint; calyx yellowish before fruiting; distributed in Mexico and Guatemala	***Spathacanthus parviflorus***
–	Corolla yellow, 31–80 mm long, throat 5–20 mm in diameter near midpoint; calyx green before fruiting; distributed in Mexico, Guatemala and Honduras	***Spathacanthus hahnianus***

#### Spathacanthus hahnianus

Taxon classificationPlantaeLamialesAcanthaceae

Baill., Hist. pl. 10: 444. Jan-Feb 1981

F7330648-B607-5293-A55C-22243A6BE683

Figs 2 , 3

Macfadyena simplicifolia Donn. Sm., Bot. Gaz. 16:198 (1891). Type: Guatemala. Alta Verapaz: borders of forest in Pasamalá, 3800 ft a.s.l., Aug 1886, von Tuerckheim 1030 (holotype: US!; isotypes: GH!, K!, M!, US!).
Spathacanthus donnell-smithii Lindau ex Donn. Sm., Bot. Gaz., 20: 293 (1895), nom. nov. superfl.
Spathacanthus donnell-smithianus Lindau, Bull. Herb. Bossier 3: 371 (1895), nom. superfl.
Spathacanthus simplicifolius (Donn. Sm.) Lindau ex Bureau & K. Schum. In C. Martius, Fl. Bras. 8: 294 (1897), basionym M. simplicifolia.

##### Type.

Mexico. Veracruz: Misantla, forêt de montagne, Santa Rita, 3 Jul 1866, L. Hahn 349 (holotype: P!).

##### Description.

Small trees or shrubs, up to 10 m height, branched, internodes glabrous. Stems quadrate to compressed when young, pubescent at nodes with eglandular trichomes. Leaves petiolate, petioles 5–45 mm long, blades elliptic to obovate-elliptic, 40–260 mm × 10–143 mm, apically acute-to-acuminate, basally acute, margin entire, adaxial surface glabrous, abaxial surface glabrous to pubescent along main veins with eglandular trichomes, flattened to flexuous. Inflorescences terminal, rachis nearly glabrous or pubescent with eglandular trichomes; bracts triangular to subulate, 1.5–6 mm × 1–2.3 mm, abaxial surface nearly glabrous or pubescent like rachis; bracteoles triangular, subulate to linear-lanceolate, 1.6 mm × 0.8–1.5 mm, abaxial surface nearly glabrous or pubescent like rachis. Flowers subsessile to pedicellate, pedicels 2–8 mm long, glabrous; calyx green, 20–40 mm × 8–19 mm, abaxially glabrous, unequally divided into 2 prominent elliptic to ovate segments, 16–30 mm long on posterior side and 3–15 mm long on anterior side; the anterior segment bilobed, lobes triangular, 0.4–2 mm long; the posterior segment trilobed, triangular lobes, 0.4–4 mm long, acute to apiculate; corolla yellow, 31–80 mm long × 20–40 mm wide, externally glabrous and internally pubescent, throat 19–33 mm long × 5–20 mm in diameter near midpoint, upper lip 12–19 mm long, lobes elliptic, (5)9–10.5 mm × 4.5–8.5 mm, lower lip 15–18 mm long, lobes linear-elliptic to elliptic, 8–15 mm long × (3.5–)7–8.5(–11) mm. Stamens whitish, longer pair 16–18.5 mm long from the base to the apex of the thecae, shorter pair 12–14.5 mm long; anthers dithecous, thecae 3.3–4.2 mm long; style glabrous 28–35 mm long; stigma lobes 0.7 mm long. Capsule 45–70 mm long, stipe 25–37 mm long, head 20–33 mm long. Seeds subcircular to subcordate, 5–10 mm long, 5.7–8 mm wide, surface roughened.

**Figure 2.**
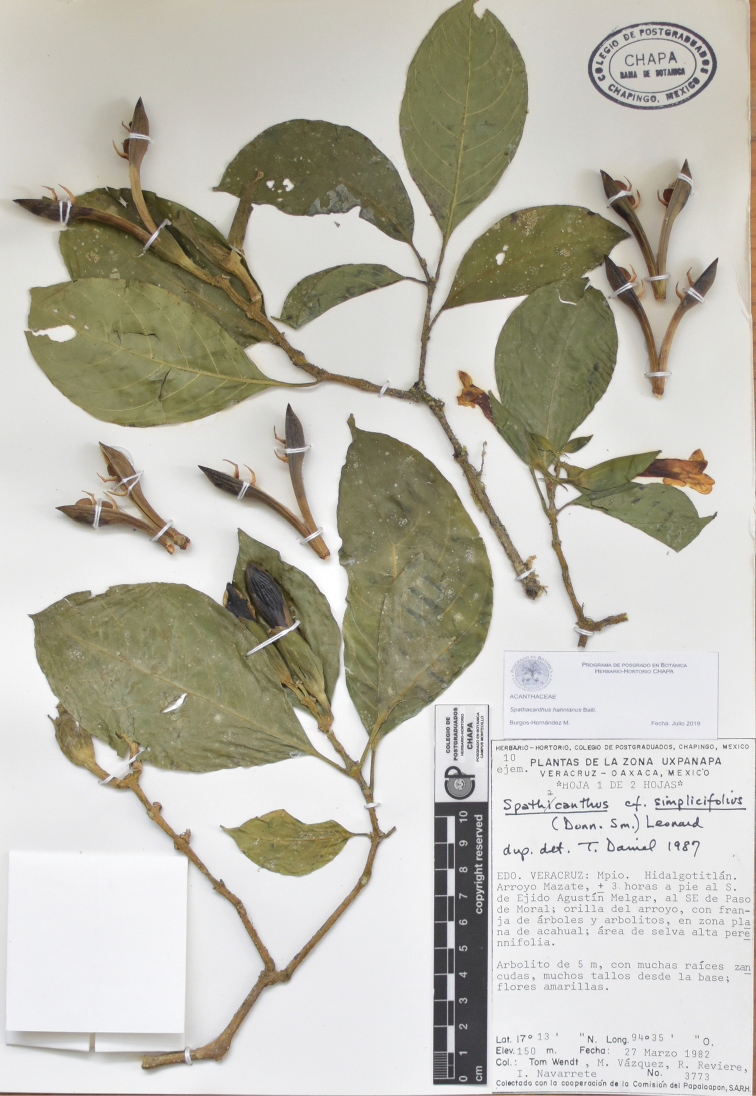
*Spathacantus hahnianus* Baillon, *T. Wendt*, *M. Vázquez, R. Reviere & I. Navarrete 3773* (CHAPA), Mexico: Veracruz, Hidalgotitlan. Note that specimen was identified as S. cf simplicifolius, a name that is synonymous of *S. hahnianus*.

**Figure 3.**
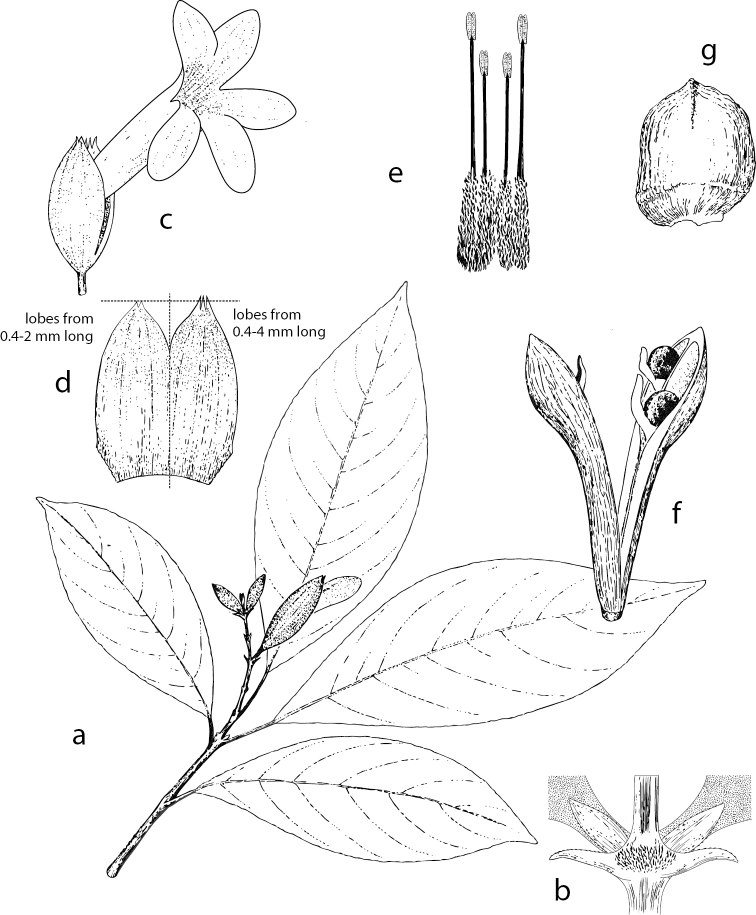
*Spathacantus hahnianus* Baillon. Image and legend modified from Daniel (1995) Flora of Chiapas, Part 4, page 131 **a** habit, × 0.5 **b** inflorescence node, × 3.5 **c** flower, × 1.1 **d** calyx split, × 1.3 **e** androecium showing didynamous stamens, × 1.8 **f** capsule, × 1 **g** seed, × 3.9. Illustration: Ellen del Valle.

##### Distribution, habitat and phenology.

*Spathacanthus hahnianus* occurs in southern Mexico (Chiapas, Oaxaca, Puebla, Veracruz), Guatemala (Alta Verapaz, Quinché) and northern Honduras (Cortés, Lempira, Yoro) (Fig. 1A). It inhabits humid low to middle elevation forests and oak forests in flooded plains, near streams and in ravines, at 100 to 2000 m a.s.l. Flowering takes place all year round, fruiting from November to June.

##### Specimens examined.

**Mexico. Chiapas**: Mpio. Berriozábal, 13 km N of Berriozábal near pozo Turipache and finca El Suspiro, 1000 m a.s.l., 02 Nov 1971, D.E. Breedlove & A. Smith 21618 (DS, DUKE, F, MEXU, MO, NY, TEX); 900 m a.s.l., 25 Dec 1972, D.E. Breedlove & R. Thome 30868 (ENCB, MO); a 12 km N de Berriozábal, 600 m a.s.l., 16 May 1989, E. Martínez & M. Soto 24241 (MEXU); La Aduana, cerca de rancho Flor de Corazón, hacia El Cairo, 800 m a.s.l., 06 Sep 1990, E. Palacios E. 1726 (IBUG, MEXU); a 13 km al N de Berriozábal, 29 Mar 1984, O. Téllez V. et al. 7598 (MEXU); Mpio. La Concordia, camino de El Triunfo para la Finca Prusia, 1940 m a.s.l., 14 May 1982, J.I. Calzada 8935 (IBUG, MEXU, XAL); Mpio. Ángel Albino Corzo, sendero Palo Gordo, Reserva de la Biosfera El Triunfo, 2000 m a.s.l., 14 May 2004, F. González-García s.n. (XAL). **Oaxaca**: Mpio. San Felipe Usila, campamento cerro Verde, carr. para arroyo Tambor, 450 m a.s.l., 02 Nov 1990, J.I. Calzada et al. 16596 (MEXU); Mpio. Santiago Lachiguiri, Distr. Tehuantepec, cerro de Buenavista, 2 km SO de crucero Buenavista, 27 Oct 1991, A. Campos V. & R. Torres 4103 (CHAPA, MEXU); Mpio. Santa María Chimalapa, Uxpanapa region, between Esmeralda (17 km E of Sarabia) and Río Verde, 1.1 mi S of Esmeralda, 100 m a.s.l., 10 Jan 1987, T.B. Croat & D.P. Jhmnon 63303 (BM, ENCB, MO, TEX); Mpio. Sta. María Chimalapa, arroyo Matzpac, N de Sta. María por la vereda al Río Verde, 250 m a.s.l., 29 Oct 1985, H. Hernández G. & C. González L. 1777 (MEXU, MO, TEX); Río Verde por la vereda a la cabecera, ca. 7 km N de Sta. María, 280 m a.s.l., 21 Nov 1985, H. Hernández G. & C. González L. 1855 (CAS, MEXU, MO, TEX); Mpio. San Felipe Usila, Nuevo Santa Flora, 22 Nov 1993, R. de Santiago & A.M. Hanan 247 (MEXU); Mpio. Guevea de Humboldt, Distr. Tehuantepec, recorrido La Cumbre-arroyo Seco, 13.4 km N de Guevea de Humboldt, 29 Mar 1991, R. Torres C. & A. Campos V. 13897 (CHAPA, MEXU); Mpio. Matías Romero Avendaño, en la estación del río Azul a 16.6 km al E de la Colonia Cuahutémoc, 1500 m a.s.l., 23 Jan 2003, E. Martínez 36091 (MEXU). **Puebla**: Mpio. Ahuacatlán, 4.5 km al SE de Ahuacatlán, brecha a Zapotitlán, 1250 m a.s.l., 24 May 1986, P. Tenorio L. et al. 11413 (MEXU); Mpio. Cuetzalan del Progreso, Tzitzinapan, Yancuictlalpan, 11 Jul 1981, F. Basurto & R. Patron 454 (MEXU); Mpio. Hueyapan, cerca de Atexcaco, 1300 m a.s.l., 12 Jul 1953, D. Gold 324 (MEXU); Mpio. Xochitlán de Vicente Suárez, 1 km al E de Pahuata, camino a Huahuaxtla, 1150 m a.s.l., 05 Aug 2014, J.L. Contreras 5604 (XAL); **Veracruz**: Mpio, Atzalan, La Calavera, 1000 m a.s.l., 07 Jul 1975, F. Ventura A. 11601 (ENCB, IEB, MEXU, XAL); La Calavera, puente La Calavera, km 12 carretera Atzalán-Tlapacoyan, 1010 m a.s.l., 08 Jul 2008, T. Krömer & J. Viccon-Esquivel 3495 (MEXU, MO, XAL); Mpio. Coatepec, Barranca de Ramírez, 1500 m a.s.l., 28 Jun 1990, P. Zamora C. 2540 (IEB, XAL); Mpio. Jesús Carranza, 3 km al este de río Chalchijapa, por la carretera Sarabia-Cedillo, 09 Jan 1975, M. Vázquez T. 1584 (IEB, XAL); Mpio. Juchique de Ferrer, cerro de La Botella, parte mediana San Alfonso, 847 m a.s.l., 24 Jul 2008, M. Vázquez T. 8635 (XAL); Mpio. Uxpanapa, km 4 camino Cedillo-La Escuadra, 150 m a.s.l., 06 Dec 1974, J. Dorantes et al. 3766 (ENCB); km 4 del camino Hnos. Cedillo-La Hulera, 150 m a.s.l., 21 Jan 1975, J. Dorantes et al. 4058 (ENCB, IEB, XAL); río Soloxúchil, 1.5 km O del campamento Hnos. Cedillo, 150 m a.s.l., 02 Jan 1975, M. Vázquez et al. 1611 (ENCB, IEB, MEXU, XAL); río Uxpanapa, cerca del límite con Oaxaca, 180 m a.s.l., 27 Sep 1980, T. Wendt et al. 2769 (CHAPA, IEB, MEXU, MO, TEX, XAL); arroyo Mazate al S de ejido Agustín Melgar, al SE de Paso de Moral, 150 m a.s.l., 27 Mar 1982 T. Wendt et al 3773 (CHAPA, MEXU, MO, TEX); 4.5 km O de Uxpanapa, sobre terracería a La Laguna, 120 m a.s.l., 17 Oct 1983, T. Wendt & I. Almaraz G. 4194 (CHAPA, MEXU, MO, NY, TEX, XAL); 1.5 km N del Poblado Dos, ejido F.J. Mina, 180 m a.s.l., 02 Feb 1983, O. Zambrano C. 1184 (CHAPA); Mpio. Yecuatla, entre La Unión y Roca de Oro, 900 m a.s.l., 21 Aug 1989 C. Gutiérrez B. 3584 (IEB, MEXU, XAL). **Guatemala. Alta Verapaz**: Pasmala, 1159 m a.s.l., Aug 1886, H. Von Tuerckheim 1030 (MEXU); **Quiché**: Chajul, La Perla, E. Tribouillier & I. Pedro 409 (BIGU); Chajul, La Perla, E. Tribouillier & I. Pedro 436 (BIGU); Chajul, aldea Chel, E. Tribouillier & I. Pedro 469 (BIGU). **Honduras. Cortés**: 2 km NW de la quebrada de Cantiles, 1700 m a.s.l., 26 Sep 1993, C. Nelson et al. 16631 (MO, TEFH). **Lempira**: Parque Nacional Montaña de Celaque, cerro Aguacatal. Las Chimis, San Manuel Colohete, P. House et al. 185 (EAP). **Yoro**: ca. 16 km from Yaruca on Quebrada de Oro to cerro Búfalo, W. Holmes 4392 (NY, TEX).

#### Spathacanthus hoffmannii

Taxon classificationPlantaeLamialesAcanthaceae

Lindau, Bull. Herb. Boissier 3: 370. 1895

848A62EE-15B1-55D4-9D12-ADAC96B74B56

Figs 4 , 5

##### Type.

Costa Rica. Alajuela: prope Aguacate, Aug 1857, C. Hoffmann 842 (holotype: B destroyed, photos F!, GH!, US!).

##### Description.

Small trees or shrubs, up to 8 m height, highly branched, internodes glabrous. Stems quadrate to flattened when young, glabrous or pubescent at nodes with flexuose eglandular trichomes. Leaves subsessile to rarely short petiolate, petioles 2–5 mm long, blades elliptic to obovate-elliptic, 72–285 mm × 25–110 mm, apically acute to acuminate, basally acute, margin undulate, glabrous on both surfaces. Inflorescences terminal, sometimes axillary, rachis pubescent; bracts triangular to lanceolate, subulate, 1–8 mm × 1–1.3 mm, abaxial surface glabrous; bracteoles lanceolate to subulate, 2.5 mm × 1–1.5 mm, abaxial surface glabrous. Flowers pedicellate, pedicels 9 mm long, glabrous; calyx green before fruiting, 10–40 mm × 10–16 mm, abaxially glabrous, equally divided into 2 prominent elliptic to obovate-elliptic segments; each segment 11–30 mm long; anterior segment entire, the posterior entire to bilobed; triangular lobes, 1.5–5 mm long, acute to apiculate; corolla white, 52–110 mm long × 30–50 mm wide, externally glabrous and internally pubescent, throat 35–50 mm long × 9–20 mm in diameter near midpoint, upper lip 15–26 mm long, bilobed, lobes ovate to elliptic, 10–20 mm × 11–18 mm, lower lip 18–25 mm long, three-lobed, lobes ovate to elliptic, 15–20 mm × 12–16 mm. Stamens whitish, longer pair 24–34 mm long from the base to the apex of the thecae, shorter pair 18–28 mm long; anthers dithecous, thecae 4–6 mm long; style glabrous 44–59 mm long; stigma lobes 0.3–0.6 mm long. Capsule 50–89 mm long, stipe 20–40 mm long, head 22–39 mm long. Seeds subcordate, 7.5–13 mm × 7.5–11 mm, surface roughened.

**Figure 4.**
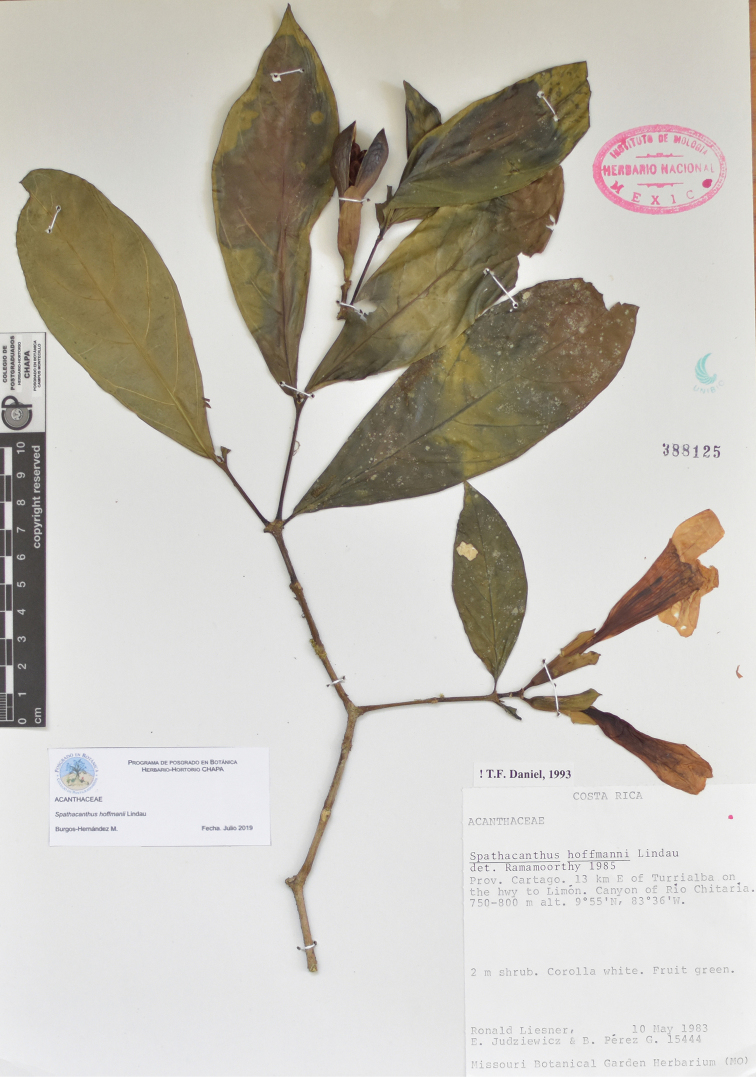
*Spathacanthus hoffmannii* Lindau. *R. Liesner, E. Judziewicz & B. Pérez G. 15444* (MEXU), Costa Rica: Cartago.

**Figure 5.**
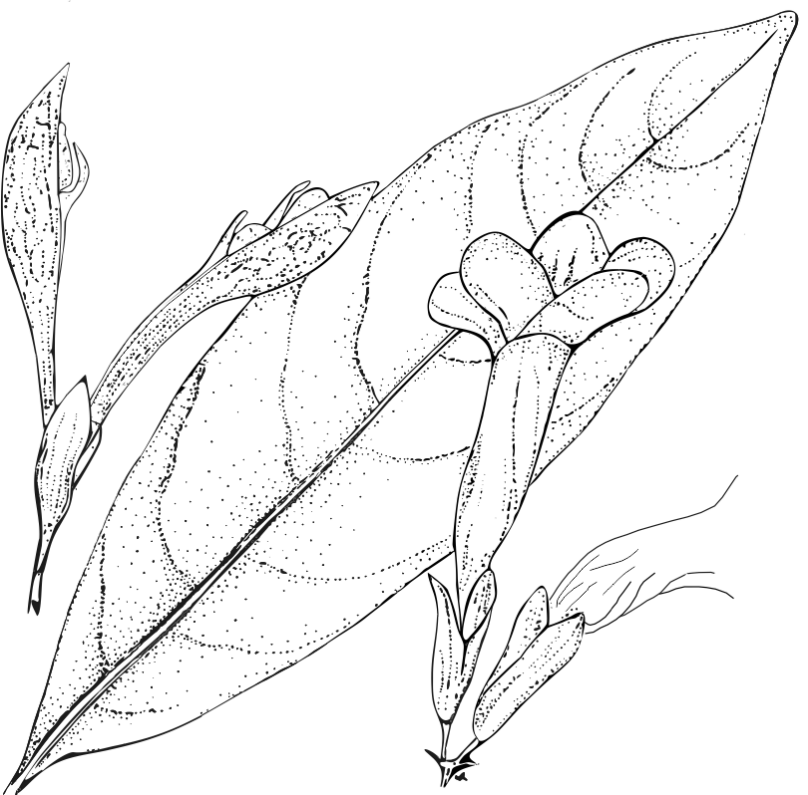
*Spathacanthus hoffmannii* Lindau. Image modified from Durkee (1986) Flora Costaricensis, No. 18, page 19. The image shows an elliptic leaf of *S. hoffmannii*, flowers with a five-lobed corolla and the 2-segments of calyx equally or subequally divided, with its large woody fruit.

##### Distribution, habitat and phenology.

*Spathacanthus hoffmannii* is known only from Costa Rica where it has been reported from all provinces (Fig. 1A). It occurs along the banks of watercourses and on cliffs in humid forests, rain forests and cloud forests at 350 to 1750 m a.s.l. Flowering takes place from January to October, fruiting from December to September.

##### Specimens examined.

**Costa Rica. Alajuela**: Est. Biol. Reserva Forestal de San Ramón, valley of Río Lorencito, Burger et al. 12439 (F); Bajos de Jamaical, Reserva de San Ramón, I. Chacón 1768 (CR, DUKE); finca Los Ensayos ca. 11 mi NW of Zarcero, 900 m a.s.l., 15 Aug 1977, T.B. Croat 43516 (MO); San José de Naranjo, I. García s.n. (CR, F); río San Lorencito, límite E de la Reserva Forestal de San Ramón, J. Gómez-Laurito 10263 (CR, F); Est. Biol. Res. Forestal de San Ramón, valley of río Lorencito on Caribbean slope, J. Gómez-Laurito & K. Swangel 12439 (CR); Reserva Forestal de San Ramón, río San Lorencito, 800–1000 m a.s.l., 05 Dec 1983, G. Herrera 362 (CR, MEXU, MO, US); Buena Vista de San Carlos, L. Holdridge 6790 (CR); Quebrada Lajas, finca Los Ensayos, Buena Vista de San Carlos, A. Jiménez M. 2319 (CR, F, MO, NY); near road to Laguna Hule, R. Lent 3243 (CR, F, MO); Cordillera de Tilarán, río La Balsa, 500 m a.s.l., 07 Mar 1994, V. Ramírez & Q. Jiménez 273 (CR, MO, NY); road to Colonia Virgen del Socorro, barranca of río Sarapiquí, 700–800 m a.s.l., 08 Aug 1979, W. Stevens 13547 (DUKE, F, MEXU, MO). **Cartago**: carretera entre Turrialba y Siquirres, a la vera del río Chitaría, J. Gómez-Laurito 6801 (CR, USJ); cerros de La Carpintera, 1600 m a.s.l., 14 Oct 1973, R, Lent 3657 (CR, F, MO); 13 km E of Turrialba on hwy to Limón, canyon of Río Chitaría, R. 750–800 m a.s.l., 10 May 1983, Liesner et al. 15444 (CR, DUKE, MEXU, MO); above Turrialtica restaurant, 36.5 km from Turrialba, R. Read & G. Daniels 74–63 (US); vicinity of Pejivalle, P. Standley & J. Valerio 46759 (F, US); 9.5 mi E of Turrialba, on rocky banks of río Chitaría, 762 m a.s.l., 12 Aug 1977, G. Webster 22253 (DUKE, F, MEXU). **Guanacaste**: El Silencio, near Tilarán, P. Standley & J. Valeria 44745 (F, US). **Heredia**: Virgen del Socorro, río Sarapiquí, Cariblanco, 600–800 m a.s.l., 31 Aug 1983, I. Chacón & G. Herrera 1211 (CAS, CR, DUKE, MO); barranca del río Sarapiquí, Colonia Virgen del Socorro, J. Gómez-Laurito 9868 (CR, F); canyon of río Sarapiquí, just upstream from bridge on rd to La Virgen del Socorro, 05 Aug 1983, B. Hammel 13304 (CR, DUKE, F, MO); camino a la Colonia de la Virgen del Socorro, rumbo a Pto. Viejo, L. Poveda 985 (CR); Vara Blanca de Sarapiquí, N slope of Central Cordillera, 1500–1750 m a.s.l., Jul 1937, A. Skutch 3325 (K, MO, NY, US). **Limón**: La Florida, voie ferrée atlantique, H. Pittier 11286 (US); río Hondo, H. Pitiier 16641 (K). **Puntarenas**: Miramar, Quebrada seca, cerro Zapotal, 18 Sep 1985, L.D. Gómez et al. 23990 (CAS, CR, DUKE, MEXU, MO). **San José**: Terrazú, Pérez Zeledón. estribaciones del cerro Diamante, 500–600 m a.s.l., 22 Sep 1998, A. Estrada et al. 1730 (MEXU); zona protectora La Cangreja, Santa Rosa de Puriscal, Q. Jiménez 482 (CR, K); zona protectora cerro Turrubares, Q. Jiménez 543 (CR).

#### Spathacanthus magdalenae

Taxon classificationPlantaeLamialesAcanthaceae

Castillo-Campos, Nordic J. Bot. 31: 449. 2013.

49757211-2286-5491-980A-FF2FB547DC7A

Figs 6 , 7

##### Type.

Mexico. Veracruz: San Andrés Tlalnelhuayocan (previously Coatepec as a publication error), Piedras Blancas, eastern slopes of the volcano Cofre de Perote, riparian vegetation, 1666 m a.s.l., 7 Mar 2012. G. Castillo-Campos et al. 27235 (holotype XAL!; isotypes ENCB!, MEXU!).

##### Description.

Small trees or shrubs, up to 12 m height, highly branched, internodes glabrous. Stems quadrate to flattened when young, glabrous or pubescent at nodes with eglandular trichomes. Leaves with petioles 10–50 mm long, blade elliptic to obovate-elliptic, 110–280 mm × 42–129 mm, apically acute to acuminate, basally acute, margin undulate, tomentose when very young along veins, then glabrous on both surfaces. Inflorescences terminal, rarely axillary, rachis glabrous; bracts triangular to subulate, 7 mm × 1 mm, abaxial surface tomentose; bracteoles triangular to subulate, 3.5 mm long, abaxial surface tomentose. Flowers subsessile to short pedicellate, pedicels to 6 mm long, glabrous; calyx yellowish before fruiting, turning green in ripe fruit, 18–25 mm × 13 mm, equally divided into 2 prominent elliptic to ovate segments; anterior segment bilobed, the posterior segment trilobed; lobes triangular, 15 mm long, apically acute to apiculate; corolla white, 58–100 mm long × 38–44 mm wide, externally glabrous and internally pubescent, throat 10–12 mm long × 3–6 mm in diameter near midpoint, upper lip bilobed, oblong, lower lip trilobed, both lobes 10–17 mm long, glabrous. Stamens whitish, longer pair about 16 mm long from the base to the apex of the thecae, shorter pair about 10 mm long; anthers monothecate, 3.5 mm long; style glabrous, 26 mm long, stigma lobes 0.3–0.7 mm long. Capsule 65–89 mm long; stipe 35–49 mm long, head 30–40 mm long. Seeds subcircular to subcordate, 8 mm × 9 mm long, surface roughened.

**Figure 6.**
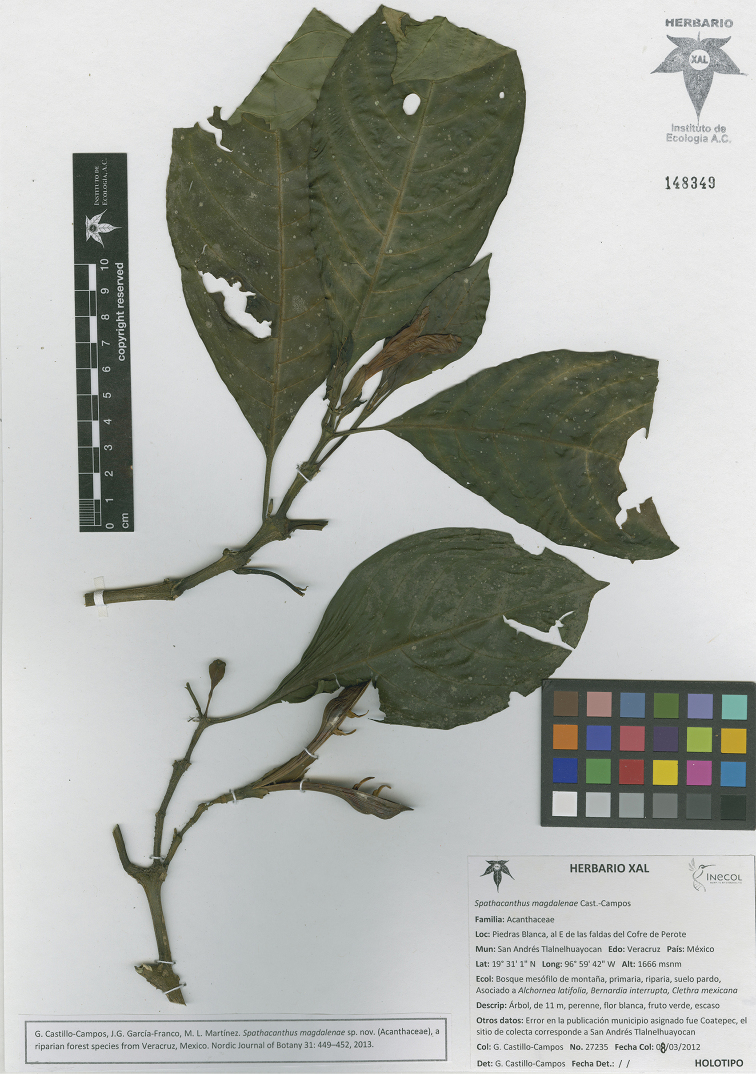
*Spathacanthus magdalenae* Castillo-Campos. *G. Castillo-Campos 27235* (XAL), Mexico: Veracruz, San Andrés Tlalnelhuayocan.

**Figure 7.**
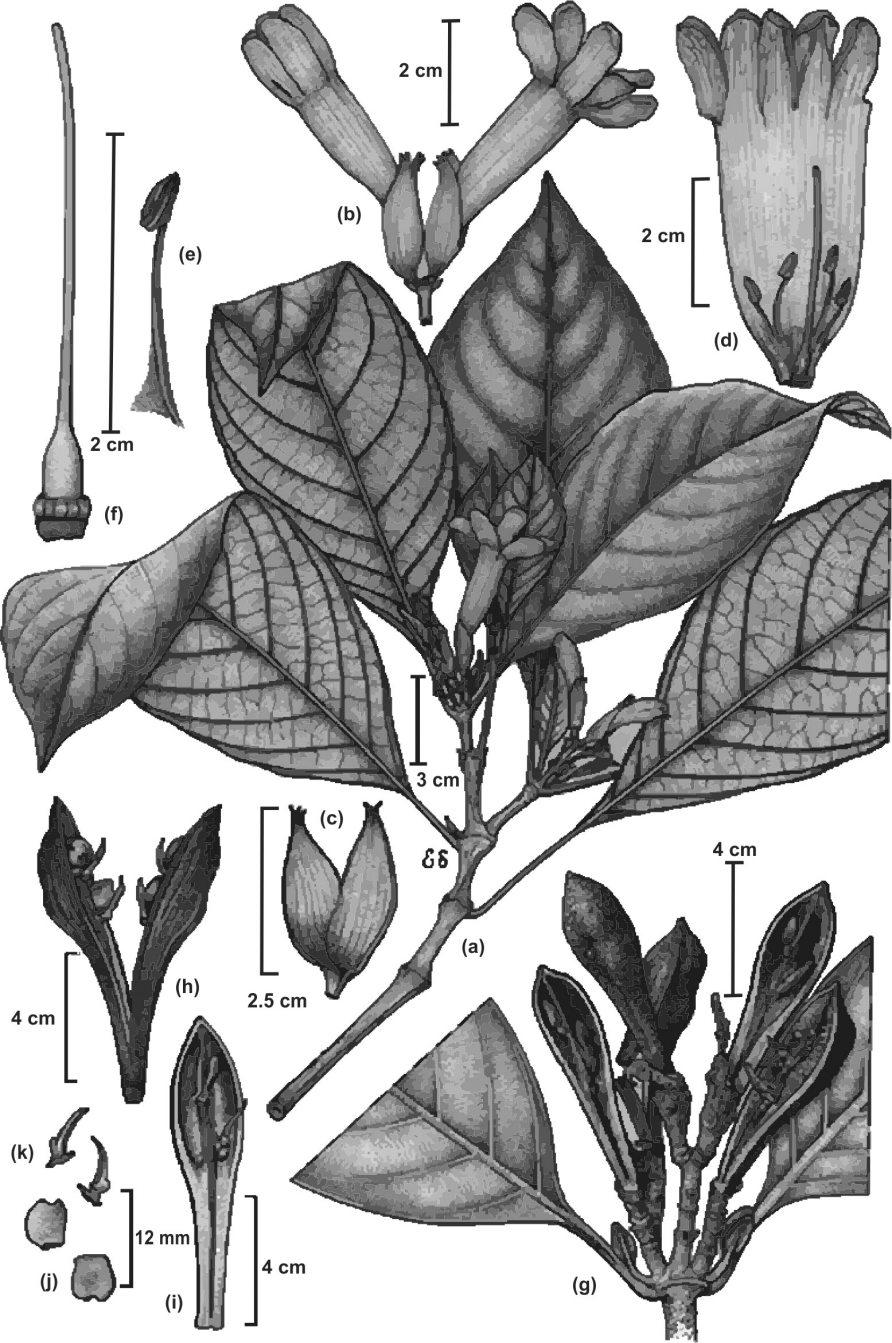
*Spathacanthus magdalenae* Castillo-Campos. Image and legend modified from Castillo-Campos et al. (2013), page 450. **a** Branch showing insertion of the leaves and position on the branch, inflorescences with flowers and buds **b** flowers, **c** calyx **d** open flower with stamens and style **e** stamen **f** style **g** branch with open capsules **h** open capsule with retinacula subtending seeds in both valves of fruit **i** interior of half of a capsule **j** seeds **k** retinacula removed from the capsules. Illustration: E. Saavedra.

##### Distribution, habitat and phenology.

*Spathacanthus magdalenae* is endemic to southern Mexico where it is restricted to the riparian vegetation of central Veracruz (Fig. 1B). It is frequent near rivers or humid canyons in cloud forests and oak forests in tropical to temperate zones, at 1300 to 1700 m a.s.l. Flowering takes place from November to March; mature fruits can be found from January to March.

##### Specimens examined.

**Mexico. Veracruz**: Mpio. San Andrés Tlalnelhuayocan, Piedras Blancas, Eastern slope of the volcano Cofre de Perote, 1666 m a.s.l., 7 Mar 2012, G. Castillo-Campos et al. 27189 (XAL, MEXU, ENCB); Piedras Blancas, Eastern slope of the volcano Cofre de Perote, 1666 m a.s.l., 30 Apr 2012, G. Castillo-Campos et al. 27377 (XAL, MEXU, ENCB); San Antonio, 1350 m a.s.l., 06 Feb 1982, F. Ventura A. 19361 (ENCB, IEB, XAL); Mpio. Jacomulco, barranca de Actopan, road to Buena Vista, 1 km after Jalcomulco, 646 m a.s.l., 12 Jun 1991, G. Castillo-Campos et al. 8206 (XAL).

#### Spathacanthus parviflorus

Taxon classificationPlantaeLamialesAcanthaceae

Leonard, Proc. Biol. Soc. Washinton 50: 15–16. 1937.

1EC2D51A-BA9E-5402-8D02-06A9EA3D4758

Figs 8 , 9

##### Type.

Guatemala. Quetzaltenango: in heavy forest of volcán Zunil, 1750 m a.s.l., 7 Aug 1934. A. Skutch 961 (holotype US!; isotypes A!, BM!, L!, NY!, US!).

##### Description.

Small trees or shrubs, up to 8 m tall, branched, internodes glabrous or nearly glabrous. Stems subquadrate to somewhat compressed when young, nodes sparsely pubescent with rigid to flexible eglandular trichomes. Leaves petiolate, petioles 5–45 mm long, blade elliptic to obovate-elliptic, 20–155 mm × 11–100 mm, apically acute to acuminate, basally acute, marginally entire, both surfaces glabrous or with eglandular trichomes along main veins, these conspicuous on abaxial surface. Inflorescence terminal, rarely axillary, rachis glabrous to pubescent with eglandular trichomas; bracts triangular to subulate, 1.3–6 mm × 0.8–1.4 mm, abaxial surface glabrous or pubescent like rachis; bracteoles triangular to subulate, 1–4 mm × 0.6–1 mm, abaxial surface glabrous or pubescent like rachis. Flowers pedicellate, pedicels 1–13 mm long, glabrous; calyx yellowish before fruiting, turning green in ripe fruit, 20–30 mm × 6.5–11 mm, abaxially glabrous, unequally divided into 2 prominent lanceolate-ovate to ovate segments, 25 mm long on posterior side and 7–17 mm on anterior side; anterior segment bilobed, lobes triangular, 0.3–0.7 mm long; the posterior segment trilobed, triangular lobes, 0.6–0.8 mm long; corolla white, 25–30 mm long × 15–25 mm wide, externally glabrous and internally pubescent, throat 12–18 mm long × 2–6 mm in diameter near midpoint, upper lip 2–3 mm long, lobes rounded, 1.5–3 mm × 1.5–2 mm, lower lip 2.5–3.5 mm long, lobes rounded, 1.3 mm × 1.5–2.5 mm. Stamens whitish, longer pair 11–15 mm long from the base to the apex of the thecae, shorter pair 8–12 mm long; anthers dithecous, thecae 3–4.5 mm long; style glabrous 10–17 mm long; stigma lobes, 0.4–0.9 mm long. Capsule 37–60 mm long, stipe 20–35 mm long, head 17–25 mm long. Seeds subcircular to subcordate, 6–10 mm long, 6–8 mm wide, surface roughened.

**Figure 8.**
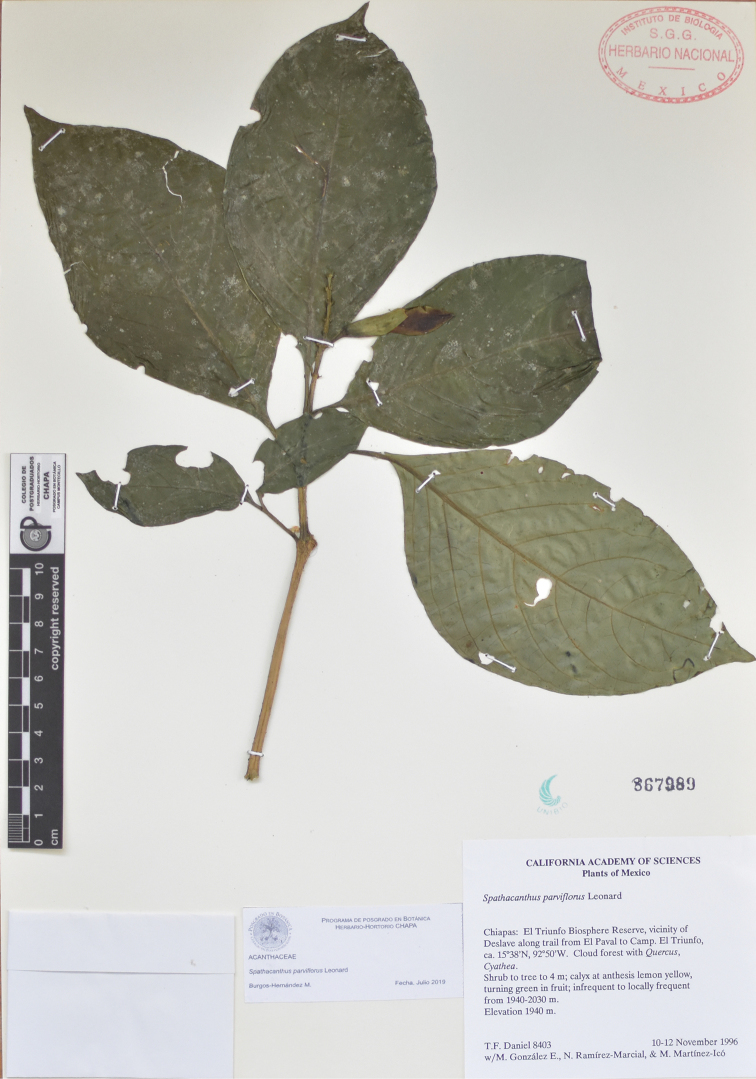
*Spathacanthus parviflorus* Leonard. *T.F. Daniel 8403* (MEXU), Mexico: Chiapas, Reserva de la Biosfera El Triunfo.

**Figure 9.**
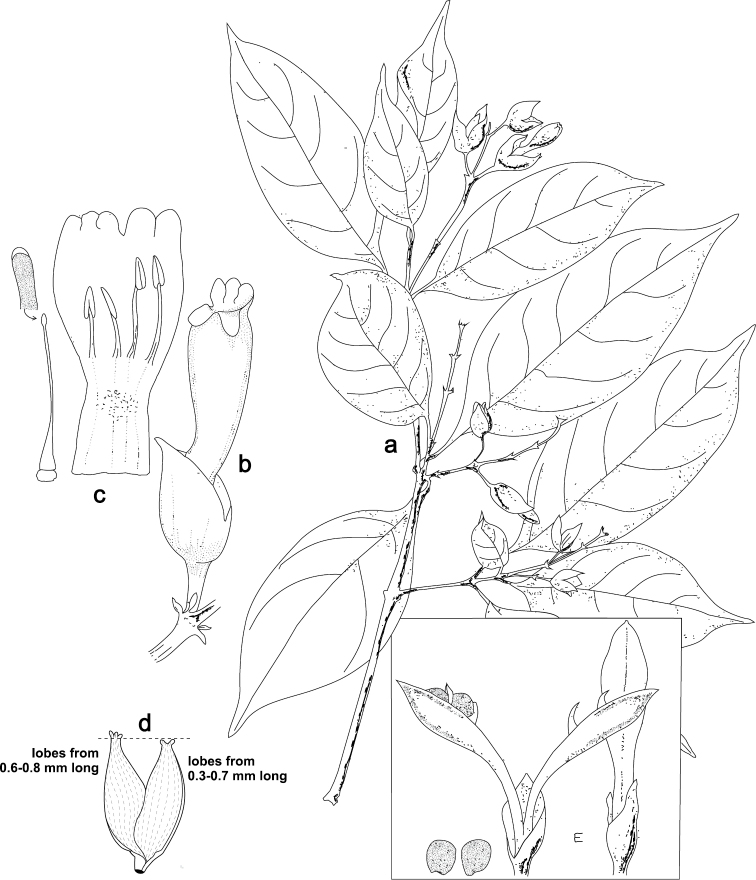
*Spathacanthus parviflorus* Leonard. Image and legend modified from Gibson (1974) Flora of Guatemala 24, part X, page 446. **A** Habit, × ½ **B** flower with bracts and bracteoles, × 1 ½ **C** corolla opened, with pistil removed, to show didynamous stamens, × 1 ½ **D** opened calyx, × 1 ½ **E** capsules with calyces, one opened to show retinacula and seeds, × 1.

##### Distribution, habitat and phenology.

*Spathacanthus parviflorus* occurs in Mexico (Veracruz, Chiapas) and Guatemala (Chimaltenango, Quetzaltenango, San Marcos, Sololá and Suchitepéquez) (Fig. 1B), along streams and flooded areas, mainly in cloud forest, less frequently in other humid forests. It has been collected at 1000 to 2300 m a.s.l. Flowering takes place throughout the year, with ripe fruits from September to February.

##### Specimens examined.

**Mexico. Chiapas**: Mpio. Mapastepec, El Triunfo Biosphere Reserve, along trail from El Paval to camp. El Triunfo, 1940–2030 m a.s.l., 10 Nov 1996, T. Daniel et al. 8403 (ENCB, K, MEXU, MO, NY, US); Mpio. Mapastepec, El Triunfo Reserve. Trail NNW from El Triunfo camp toward Palo Gordo Camp., 1–3 km from El Triunfo camp. El Triunfo Reserve, 2000 m a.s.l., 21 Feb 1990, R.J. Hampshire et al. 522 (MEXU); Reserva El Triunfo (campamento/HQ), near campamento El Triunfo, 2000 m a.s.l., Sep 1989, M. Heath & A. Long MA84 (CHIP, MEXU); Reserva El Triunfo, polígono 1, campamento /HQ - finca Prusia, 1900 m a.s.l., 11 Dec 1989, M. Heath & A. Long 491 (CHAPA, MEXU); between Cañada Honda and El Triunfo, slopes of sierra de Soconusco, 1300–2100 m a.s.l., 06 Nov 1945, E. Xolocotzi & A. Sharp 338 (DS, MEXU); Reserva de la Biosfera El Triunfo, polígono zona núcleo I, 1800 m a.s.l., 06 Aug 2005, N. Martínez 1149 (MEXU); Reserva El Triunfo, Palo Gordo-finca Catarrinas, M. Heath et al. 738 (CHIP); Mpio. La Concordia, camino entre finca Custepec and San Antonio Miramar (pass), M. Heath & A. Long 834 (CHIP); Reserva de la Biosfera El Triunfo, N. 1850 m a.s.l., 16 Jun 1994, Ramírez-Marcial & P. Quintana-Ascencio 507 (CAS, MEXU); Mpio. Undetermined, Mt. Pistar, 03 Aug 1937, E. Matuda 1696 (F, MO, NY, UT, US). **Veracruz**: Mpio. Uxpanapa, Arroyo Zarco, 15 km al S de La Laguna, 200 m a.s.l., 24 Nov 2012, G. Castillo-Campos & L. Aragón A. 27651 (XAL). **Guatemala. Chimaltenango**: SW slope of volcán Fuego above finca Montevideo, J. Steyermark 52100 (F, US). **Quezaltenango**: 1400–2250 m a.s.l., 14 Jun 2005, M. Pérez 563 (MO); Pacific escarpment, 3 km S of Santa María Planta eléctrica on Hwy 9S, K. Roe et al. 715 (BM, US); volcán Zunil, 1737 m a.s.l., 07 Aug 1934, A. Skutch 961 (F, IEB); along Quebrada San Gerónimo, finca Pirineos, lower S-facing slopes of volcán Sta. María, between Sta. María de Jesús and Calahuaché, J. Steyermark 33359 (F); lower S-facing slopes of volcán Santa María, between Santa María de Jesús and Calahuaché, J. Steyermark 33507 (F). **San Marcos**: La Trinidad, ca. 2 km from finca Armenia above San Rafael, 1100–1250 m a.s.l., 12 Jul 1977, T.B. Croat 40846 (CAS, MEXU, MO); finca Armenia, San Rafael Pie de la Cuesta, 1524 m a.s.l., 06 Jul 1977, J.D. Dwyer 14412 (CAS, MO); finca Armenia, San Rafael Pie de la Cuesta to Carrizal, past finca África, 1300–1600 m a.s.l., 09 Aug 1980, J.D. Dwyer 15340 (MEXU, MO); volcán Tajumulco, above finca Porvenir on “Todos Santos Chiquitos”, J. Steyermark 37204 (F). **Sololá**: sin localidad, 1000 m a.s.l., Aug 1993, M. Flores, 27 (MO); finca Mocá, Guatalón, S slope of volcán Atitlán, W. Hatch & C Wilson 342 (F). **Suchitepéquez**: Sta. Bárbara, finca Sta. Adelaida, K. Lind 69 (F); volcán Sta. Clara, between finca El Naranjo and upper slopes, J. Steyermark 46632 (CAS, F, NY, US).

### Phylogenetic relationships

**Figure 10.**
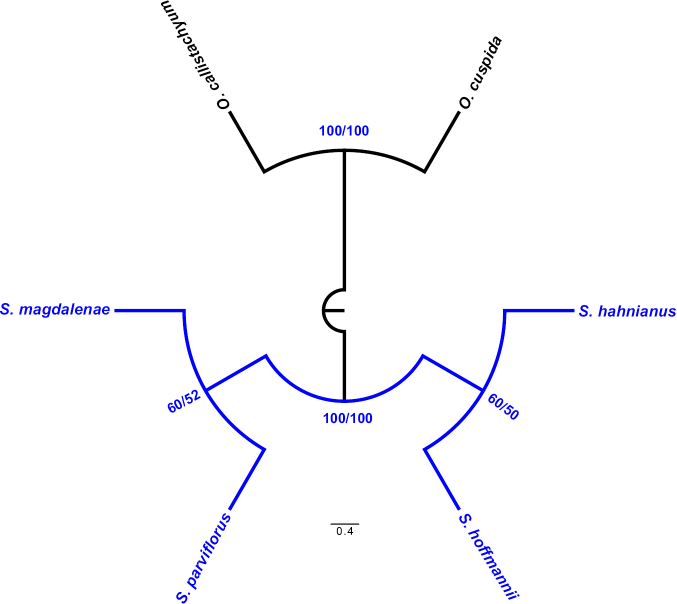
Majority-rule consensus tree inferred from parsimony analysis of 15 morphological characters for four species of *Spathacanthus* (tree length = 24 steps, CI = 0.88 and RI = 0.80). Bootstrap values (BS; left) and Jackknife values (JK; right) are shown above branches. Only support values above 50% are shown. Representatives of the genus *Odontonema* were used as out-group.

A total of 15 characters were analysed, 14 (93.3%) of which were parsimony-informative. The MP analysis resulted in five most parsimonious trees of 24 steps, a consistency index (CI) of 0.88 (excluding uninformative characters) and a retention index (RI) of 0.80. The majority rule consensus tree is shown in Fig. 10.

The monophyly of the genus *Spathacanthus* was strongly supported (BS and JK = 100%). Within the genus, two clades were recovered, the first one includes the species *S. hahnianus* and *S. hoffmannii* as more closely related, with moderate support (BS = 60% and JK = 50%) (Fig. 10). In the second clade, two of the white flower species, *S. parviflorus*, and *S. magdalenae* were recovered as sister taxa, with support values of BS = 60% and JK = 52%.

## Discussion

*Spathacanthus* is composed of four recognised species, all of which are restricted to Mesoamerica. *Spathacanthus hahnianus* is the most widely distributed species, spanning from southern Mexico and Guatemala to Honduras. *Spathacanthus parviflorus* occurs only in southern Mexico and Guatemala; *S. magdalenae* and *S. hoffmannii* are geographically restricted, the first as micro-endemic to the centre of Veracruz, Mexico and the second as endemic to Costa Rica, where it is widely distributed throughout that country. Narrowly distributed species like *S. magdalenae* are of conservation concern as they may be threatened by the effects of environmental deterioration and habitat loss (Castillo-Campos et al. 2005; Mooers and Redding 2009). Despite this, none of these species has been formally assessed using the IUCN Red List standard. This highlights the need for more studies on each of the species in the group that allow us to know if it is necessary to take measures for conservation.

Plants of *Spathacanthus* occur mainly in tropical rainforests and cloud forests and at elevations between 100 and 2,300 m a.s.l. Of the four species, *S. hahnianus* and *S. hoffmannii* thrive in areas below 1,000 m a.s.l. Plants occur less commonly in higher elevation temperate forests. Particularly, *S. parviflorus* is restricted to cloud forests, *S. hoffmannii* inhabits humid tropical forests and *S. magdalenae* is usually found in cloud forests and oak forests. Meanwhile, *S. hahnianus* occurs in all of these environments. This information is relevant because, globally, montane cloud forests and tropical rainforests are threatened ecosystems. Particularly, the first is considered rare due to its restricted extent of coverage. A mere 2.5% of the total area of the tropical forests worldwide is cloud forest (Bruijnzeel et al. 2010; Sánchez-Ramos and Dirzo 2014). Three of the four species occur naturally in Mexico where tropical forests occupy only 1% of the territory; nevertheless, these ecosystems harbour a large number of species, representing 27% of the floristic richness of the country (Gual-Díaz and Rendón-Correa 2014). Worldwide, Central America is one of the regions most affected by deforestation. An important issue is that no collections of *Spathacanthus* have been made from Nicaragua or El Salvador. It is not clear whether these countries remain inadequately known botanically or whether plants of *Spathacanthus* are genuinely absent from these countries. Based on proximity and shared climate and vegetation types, further botanical exploration is warranted.

The detailed taxonomic review of the four species revealed a number of specimens that were misidentified. For example, Daniel (1999) noted that a white corolla had been reported for *S. hahnianus* by *Ventura A. 19361* (XAL, IEB). However, this specimen corresponds to *S. magdalenae* (revised and corrected in this study). Other records of plants with white corollas, such as *L.D. Gómez et al. 23990* (MEXU) and *A. Estrada et al. 1730* (MEXU) from Costa Rica, correspond to *S. hoffmannii*. Specimens of *S. hahnianus* (e.g. *C. Gutiérrez B. 3584* (IEB, MEXU, XAL); *J. Dorantes 3766* (XAL), *4058* (ENCB, IEB, XAL); *M. Vázquez T. 1584* (IEB, XAL); *J.I. Calzada 8935* (IBUG, MEXU, XAL); *F. Gónzalez-García s.n*. (XAL)) have been misidentified as *S. parviflorus*. As species are circumscribed here, intraspecific variation in corolla colour can be ruled out (see Daniel 1999 for discussion). This study demonstrates corolla colour is a key morphological character for differentiating species (see the identification key above). However, since the work by Daniel (1999), no taxonomic has been carried out on *Spathacanthus*, such that such errors were perpetuated. Consequently, we also find labels with scientific names that are no longer valid today. In addition, our study found that only *S. magdalenae* has monothecate anthers, an autapomorphy for the species. Therefore, this and other slight modifications were made in the description of the genus, since it was originally described as having dithecate anthers only. These results highlight the importance of taxonomic research as we report here.

Until recently, *S. hahnianus* was known only from Mexico and Honduras. Daniel (1999) reported a single specimen of *Spathacanthus* from Guatemala but the condition of the specimen did not allow it to be identified. A decade later, Daniel (2010) suggested that the species was either rare or extirpated in Guatemala. In 2012, in a report on new distribution records of Acanthaceae in Guatemala, the same author noted the presence of the species in the montane cloud forests of Quiché. Similarly, in Honduras, the species was only known from Yoro, but is here reported also from the provinces of Cortés and Lempira. Thus, this species is now known to range more widely in Guatemala and Honduras than had been previously recognised.

### Phylogenetic analysis

The parsimony phylogenetic analysis retrieved *Spathacanthus* as a monophyletic group, which is consistent with the results of Daniel (1999). In his previous cladistic analysis, *S. hoffmannii* was more closely related to *S. hahnianus* because both species have long corollas and the lower lip ends in relatively long lobes. On the other hand, based on similarities and differences according to the morphological key proposed by Castillo-Campos et al. (2013), it is suggested that *S. magdalenae* was closest to *S. parviflorus*, although the former has longer flowers, fruits and seeds, as well as wider leaves, relative to *S. parviflorus*.

Our results are concordant with those suggested from both of these works. However, the basal position of *S. parviflorus*, resulting from Daniels’s analysis, changes when *S. magdalenae* is included, supporting the hypothesis, proposed by Castillo-Campos et al. (2013), that *S. magdalenae* and *S. parviflorus* are closely related species; both share white corollas (as does their sister taxon, *S. hoffmannii*) and leaves petiolate. The plants of these species are mainly distributed in the cloud forest of the Pacific regions, only above 1000 m a.s.l. For their part, *S. hahnianus* and *S. hoffmannii* share long corollas and they are the most widely distributed species in terms of habitats and altitude. *Spathacanthus hahnianus* is the most widely distributed geographically, meanwhile, *S. magdalenae* and *S. hoffmannii* have a more limited distribution on the Mexican Pacific slopes and in Costa Rica, respectively. Although the results are concordant with previous studies, it should not be forgotten that the cladistic analysis, presented here, is only exploratory, so increased character sampling and the acquisition of molecular data integrated into a framework of total evidence will support a test of the phylogenetic hypothesis proposed herein.

## Conclusions

Our analysis yielded a more in-depth insight into the distribution, characters and ecological features of plants of the Mesoamerican genus *Spathacanthus*; however, this genus remains poorly known in Mexico and Central America. Future research should specifically seek to collect plants of *Spathacanthus* species in suitable habitats across the Mesoamerican region. We expect that extensive fieldwork will reveal new areas of distribution and perhaps new species of *Spathacanthus*. Molecular data should be gathered to test the phylogenetic hypothesis presented here; notably, strategic sampling within species would shed light on the processes of speciation.

## Supplementary Material

XML Treatment for Spathacanthus

XML Treatment for Spathacanthus hahnianus

XML Treatment for Spathacanthus hoffmannii

XML Treatment for Spathacanthus magdalenae

XML Treatment for Spathacanthus parviflorus

## Appendix 1

**Table:** Morphological characters and character states used in this study.

Characters	Character states			
	0	1	2	3
Calyx color	Yellowish	Green	Purple	Reddish
Shape of divisions of the calyx	Homomorphic	Heteromorphic		
Pubescence in calyx	Glabrous	Pubescent		
Calyx length	1–9mm	13–40mm		
Type of calyx	Spathaceous	Pentamer		
Apex of calyx segments	Entire	Bifid	Trifid	
Corolla color	Yellow-yellowish	White	Pinkish-purple	Red
Corolla length	17–30mm	31–110mm		
Outer corolla surface	Glabrous	Pubescent		
Number of stamens	2	4		
Type of stamens	Homogeneous	Didynamous		
Style length	5–17mm	21–38mm	44–59mm	
Margin of leaves	Entire	Undulate		
Pedicel type	Pedicellate	Subsessile		
Capsule length	9–27mm	40–89		

## Appendix 2

**Table:** Matrix of morphological characters of *Spathacanthus* and out-groups used for the cladistic analysis.

Characters	*S. hahnianus*	*S. hoffmannii*	*S. parviflorus*	*S. magdalenae*	*O. callistachyum*	*O. cuspidatum*
Calyx color	1	1	0	0	2	3
Shape of divisions of the calyx	1	1	1	1	0	0
Pubescence in calyx	0	0	0	0	1	1
Calyx length	1	1	1	1	0	0
Number of segments in calyx	0	0	0	0	1	1
Apex of calyx segments	2	1	2	2	0	0
Corolla color	0	1	1	1	2	3
Corolla length	1	1	1	0	0	0
Outer corolla surface	1	1	1	1	0	0
Number of stamens	1	1	1	1	0	0
Type of stamens	1	1	1	1	0	0
Style length	1	2	0	1	0	0
Margin of leaves	0	1	0	1	0	0
Pedicel type	1	0	0	1	1	1
Capsule length	1	1	1	1	0	0
